# VGGFace-Ear: An Extended Dataset for Unconstrained Ear Recognition †

**DOI:** 10.3390/s22051752

**Published:** 2022-02-23

**Authors:** Solange Ramos-Cooper, Erick Gomez-Nieto, Guillermo Camara-Chavez

**Affiliations:** 1 Department of Computer Science, Universidad Catolica San Pablo, Arequipa 04001, Peru; solange.ramos@ucsp.edu.pe (S.R.-C.); emgomez@ucsp.edu.pe (E.G.-N.); 2 Computer Science Department, Federal University of Ouro Preto, Ouro Preto 35400-000, Brazil

**Keywords:** ear recognition, ear biometrics, deep learning, convolutional neural networks, mask-RCNN, VGGFace, transfer learning

## Abstract

Recognition using ear images has been an active field of research in recent years. Besides faces and fingerprints, ears have a unique structure to identify people and can be captured from a distance, contactless, and without the subject’s cooperation. Therefore, it represents an appealing choice for building surveillance, forensic, and security applications. However, many techniques used in those applications—e.g., convolutional neural networks (CNN)—usually demand large-scale datasets for training. This research work introduces a new dataset of ear images taken under uncontrolled conditions that present high inter-class and intra-class variability. We built this dataset using an existing face dataset called the VGGFace, which gathers more than 3.3 million images. in addition, we perform ear recognition using transfer learning with CNN pretrained on image and face recognition. Finally, we performed two experiments on two unconstrained datasets and reported our results using Rank-based metrics.

## 1. Introduction

Identifying people is a persistent issue in society. Different areas such as forensic science, surveillance, and security systems usually demand solutions for this issue. Most identification systems implement biometrics to fulfill their requirements. A biometric trait has unique and specific features that make people’s recognition possible. Among the most common physical biometric traits such as fingerprints, palmprints, hand geometry, iris, and face, the ear structure results in an excellent source to identify a person without their cooperation. It provides three meaningful benefits, i.e., (i) the outer ear structure does not drastically change over a person’s lifetime, (ii) it can be captured from a distance and, (iii) is unique for everyone, even for identical twins [1]. When a person ages, the ear shape does not show significant changes between 8 and 70 years [2]. After the age range of 70–79 years, the longitudinal size slightly increases while the person gets old; however, the structure remains relatively constant. Facial expressions do not affect the ear shape either. the ear image has uniform color distribution and is completely visible even in mask-wearing scenarios. Moreover, the ear structure is less prone to injuries than hands or fingers. These features make the ear structure a stable and reliable source of information used in a biometric system.

**Figure 1:**
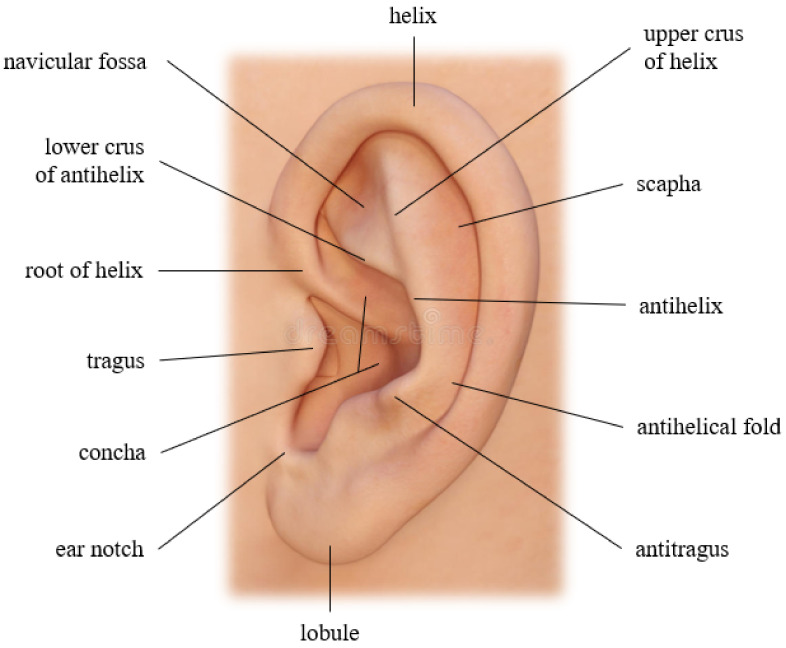
Outer ear structure, image elaborated by the author.

The appearance of the outer ear is defined by the lobule, tragus, antitragus, helix, antihelix, concha, navicular fossa, scapha, and some other critical structural parts, as Figure 1 illustrates. These anatomical characteristics differ from person to person and have also been recognized as a means of personal identification for criminal investigators. In 1890, a French criminologist named Bertillon [3] was the first to research ears and their potential for individual identification. In 1964, an American police officer named Alfred Iannarelli gathered more than 10,000 ear images and conducted some studies where he determined 12 ear characteristics to identify an individual. He discovered that even twins and triplets have different ears, concluding that ears are even unique among genetically identical subjects [4]. Even though ears have distinctive advantages over other standard biometric identifiers, their recognition has significant challenges to be addressed, especially when it comes to unconstrained settings or real-world scenarios (a.k.a. recognition in the wild). We can mention blurring, low resolution, illumination variations, and occlusion caused by hair, head, and ear accessories, as some of these issues.

Recently, approaches that apply convolutional neural networks (CNN) represent the state-of-the-art performance in tasks such as object detection, segmentation, and image classification in unconstrained settings. The submissions to the ImageNet Large-Scale Visual Recognition Challenge (ILSVRC), one of the most well-known computer vision competitions, can evidence this trend. Here, algorithms based on Deep Learning (DL) techniques show best performances on most common computer vision tasks [5]. Unlike other approaches that make use of, for example, handcrafted feature extractors to describe images, which usually perform better when input images come from within controlled conditions [6,7,8,9]. In that vein, one crucial concern when applying CNNs for recognition in the wild is what kind of data we use to train models. Using real-world-like images can build a robust feature descriptor algorithm aware of all challenges and problems present in real-world settings.

Nonetheless, the type and the amount of data available are essential features when we train models. Let us take a look at one similar task: face recognition. This task has also been approached with DL techniques, reaching a significant maturity level and benefiting from large-scale datasets proposed in the literature. For instance, the Google FaceNet [10] used 200 million images to train, the DeepFace [11] trained an architecture using 4.4 million images, and the VGGFace [12], a VGG16-based model, was trained with 2.6 million unconstrained face images. These architectures have led to some of the most exciting biometrics, security, and commercial applications.

In practice, building large-scale databases is a complex task. However, to make use of deep learning techniques appropriately in the ear recognition task, it is necessary to count on considerable amounts of training data. Leveraging publicly available face datasets for building ear datasets was also explored in [13,14]. However, the collected samples present a regional bias in the first case since they all were gathered from Chinese people. In the second case, the face dataset and ear coordinates are unavailable. In our case, using one of the most extensive face datasets available, the VGGFace2 [12], we built and proposed an extended ear dataset for training and testing approaches in the unconstrained ear recognition context. Furthermore, we apply transfer learning from pretrained models on face images to adjust and adapt them to the ear domain. According to our experiments, using face-based models to transfer learned features performs better than using ImageNet-based pretrained models.

Outcomes and major contributions of the proposed work are defined as follows:

- We prepare an unconstrained ear database named VGGFace-Ear dataset to extend the existing VGGFace dataset.
- We fine-tune pretrained models on general and face images to adapt the models to the ear recognition domain using the proposed dataset.
- We use the fine-tuned models as feature descriptors to evaluate the generalization capability of models to unknown identities using two different unconstrained ear datasets.

In a previous version of this paper [15], we fine-tune models using a reduced part of the VGGFace dataset. In this version, we present the completed extended version of the VGGFace-Ear dataset, which was used to train and test models according to the proposal. In addition, we provide an up-to-date database review available in the ear recognition field as well as describe the process of building the dataset and annotation files.

The rest of this document is organized as follows. In Section 2, we give an overview of some available datasets proposed for ear detection, segmentation, and recognition. A brief review of recent related work on ear recognition is presented in Section 3. Detailed description of the proposed dataset is provide in Section 4. In the next section, we describe the transfer learning technique employed. In Section 6, we show experimental results and analysis using the proposed dataset. Finally, conclusions and future works are mentioned in Section 7.

## 2. Ear Databases Review

This section will review some ear datasets proposed and used to train and test different approaches in the literature. The first two datasets address the ear segmentation problem. They present pixelwise annotations specifying whether a pixel belongs to the ear object or not. Following datasets are subject-annotated; thus, they are used for ear recognition and verification problems. According to the images they collected, these datasets were classified as constrained, semiconstrained, and unconstrained databases. Finally, we will mention one dataset intended for alignment and normalization problems since images present ear landmarks annotations.

Among datasets with pixelwise annotations, one of the databases is the University of Beira Interior Ear Dataset (UBEAR) [16]. This collection gathers 4430 grayscaled images of 126 subjects. Most individuals were under 25 years old. This dataset does not present tightly cropped ear region images but full face and background images of 1280×960 pixels size. in addition, it shows a corresponding binary ear mask for every single image, manually annotated. All images were taken from video recordings of all subjects facing front and 3 meters apart from the camera sideways. Then, individuals were asked to move their heads upwards–downwards, and outwards–towards. After these, subjects should step ahead and backward from the initial position. For each person, both ears were captured in two different sessions, giving a total of four videos per subject. Each video sequence was manually analyzed, selecting around 17 frames. Besides a binary mask, this dataset also presents subject annotations and left–right ears annotations. Images were stored in TIFF and BMP formats.

**Figure 2:**
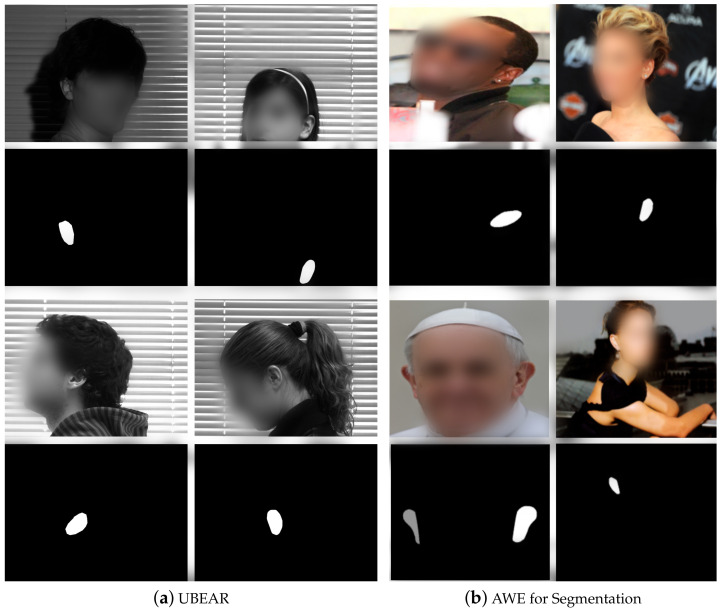
Ear segmentation datasets samples. Pictures of every person are over their own ear segmentation image (mask image). (**a**) UBEAR dataset contains images taken within the same environment and specific head positions for everyone. (**b**) AWE dataset gathers images with high variation and in some cases, the two ears are present in the picture.

Unlike the UBEAR dataset, the Annotated Web Ears (AWE) for Segmentation dataset [17,18] collects images taken under unconstrained environments since they were gathered from the web and of different resolutions. It contains 1000 images with a great variety in appearance, ethnicity, occlusion, head position, head side, and illumination. The dataset was split into two groups: training subset with 750 images and test with 250 images. Each image is 480×360 pixels size, colored, and saved in PNG format. Every image has its corresponding bounding box and pixelwise binary mask annotation image. They do not contain subject annotations. Sample images from these two datasets are shown in Figure 2.

**Figure 3:**
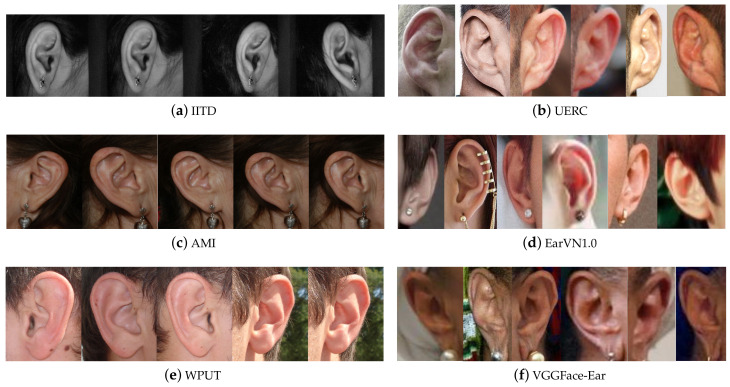
Ear datasets samples. The left column group is constrained and semiconstrained datasets (IITD, AMI, WPUT), while the right column group shows samples from unconstrained datasets (UERC, EarVN1.0, VGGFace-Ear). All samples belong to the same individual. Thus, it can be noticed the variability among samples from the same class.

Datasets that were specifically subject annotated are the following: first, the Indian Institute of Technology of Delhi (IITD) Ear Database [19]. It is a constrained set that gathers 493 images belonging to 125 subjects. Each person has from 3 to 5 right-ear image samples. Images are grayscaled, have the same size (272×204 px), and are saved in BMP format (see Figure 3a). There is not much variation in pose since all images were taken in profile face position, as well as, the use of accessories and occlusion cases are minimum. Images were acquired using a digital camera in an indoor environment over nine months and no additional illumination was involved. Each volunteer sat on a chair, and a digital camera was fixed to acquire the region of interest, ensuring the presence of the ear in the imaging window. All the subjects in the database are 14–58 years old.

Second, we have the Mathematical Analysis of Images (AMI) Dataset [20]. This constrained set collects images from students, teachers, and staff of the Computer Science Department at Las Palmas de Gran Canaria University in Spain. It contains 700 images belonging to 100 different individuals between 1–65 years old. Each subject presents seven samples, one from the right ear and six from the left ear. All images are colored, the same size (492×702 px), and saved in PNG format (see Figure 3c). Images were taken in an indoor environment under the same lighting conditions. The subject is seated about two meters from the camera and looking at some previously fixed marks. Thus, it presents a low variation in illumination and head position, but occlusion and accessories are present in some cases.

Third, we have the West Pomeranian University of Technology (WPUT) Dataset [21]. It gathers 2070 images from 501 individuals (254 women and 247 men), most of them in the range of 21–35 years old. It presents from 4 to 8 images per subject (at least two images per ear). For 451 subjects, samples were taken on only one session, while the rest had repeated sessions. This dataset was built to reflect real-life conditions and gather as much variety as possible. These variations are associated with occlusion caused by hair, earrings, earplugs, glasses, dirt, dust, head accessories, among others. the 20% of the pictures are free of any auricle occlusions, 15.6% were taken outdoors, 2% were taken in the dark. Even though images show a wide range of appearance variability, they were still acquired in laboratory-like settings and simulated the most challenging problems in the ear recognition context. Thus this could be classified as a semiconstrained dataset. All images are colored, the same size (380×500 px), and saved in JPG format (see Figure 3e).

Fourth, we have the Annotated Web Ears (AWE) Dataset [22]. This set contains 1000 images of 100 subjects. Every individual, who mostly are famous characters and public figures, presents ten images of different quality and sizes. Unlike the previous datasets, images from this group were compiled from the Web using crawlers; thus, it can be classified as an unconstrained dataset. Images are tightly cropped and annotated with an identification number, gender, ethnicity, accessories, occlusions, head pitch, head roll, head yaw, head side, and central tragus point in a JavaScript Object Notation (JSON) file. All images are colored and saved in PNG format. After some years, the authors presented one first extension, the Annotated Web Ears Extended (AWEx), which was presented in [23] and gathered 246 classes more, with a total of 4104 images. Then it was extended again and adapted for the Unconstrained Ear Recognition Challenge (UERC) [24,25], collecting 11,804 images of 3706 subjects. In this case, the dataset was divided into two parts. The training split gathers 2304 images from 166 subjects (at least ten images per subject). the smallest image size in this set is 15×29 pixels, and the biggest image is 557×1008 pixels size. On the other hand, the testing split contains 9500 belonging to 3540 subjects (with a variable number of images per subject). In this group, the smallest image is 11×21 pixels size, and the biggest image reaches 499×845 pixels size. This collection shows a high appearance variability not only among individuals but also between every image of every individual (see Figure 3b). In some cases, the difference at times the images of an individual were taken is up to many years, something that is not able to find in constrained laboratory datasets. In addition, images show variability in illumination, head position, different background, occlusion, people ethnicity, and age. Thus, images reflect real-life conditions.

Fifth, we have the EarVN1.0 Dataset [26]. This set gathers 28,412 images from 164 Asian people. This collection falls into the group of unconstrained datasets since images present high variability among samples of one class regarding illumination, head position, scale, contrast, occlusion, and the use of accessories. Nonetheless, all images belong to individuals from the same region and ethnicity, raising a bias when training a model. Each class has at least 100 samples of both left and right ears. All subjects in the dataset were volunteers, 98 were males, and 66 were females. The original facial images have been acquired in an unconstrained environment, including cameras systems and light conditions. Among all images, the smallest image is 13×17 pixels size, and the biggest image reaches 436×581 pixels size. Samples are saved in JPEG format (see Figure 3d).

**Figure 4:**
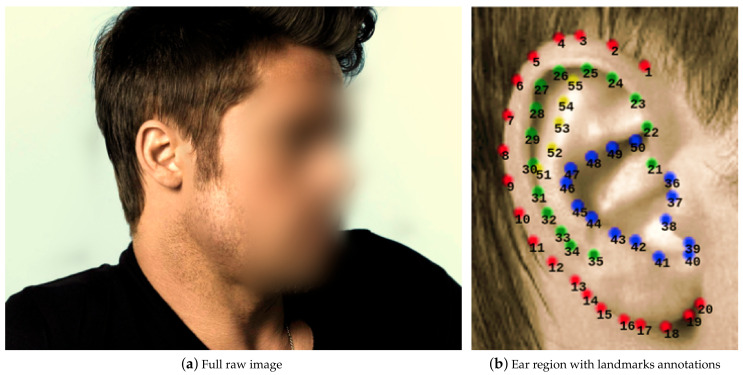
Earlandmarks distribution according to [27]. Note that landmarks from 1 to 20 (red color) define the helix’s outer part and the lobe. From 21 to 35 (green color), define the inner part of the helix. from 36 to 50 (blue color), define the concha and the antihelix. Landmarks 35 to 38 define the tragus, and landmarks 40 to 42 define the antitragus. Finally, from 51 to 55 (yellow color) define the upper crus of the helix. Images elaborated by the author.

Finally, we describe one last available database with landmarks annotations to approach the alignment and recognition problems within the ear context. The In The Wild Dataset [27] gathers 2058 images belonging to 231 subjects where all of them are public characters. This dataset contains two collections; the first collection, called A, gathers 605 images. This set was randomly divided into two disjoint sets, a train set with 500 classes and a test set with 105. The second collection, named B, has landmarks annotation and class annotated with the person’s name in the picture, containing from 1 to 14 images per class. It is worth mentioning that both collections include the same 2058 images, but they are grouped differently. All images are variable size, saved in PNG format, and corresponding text file with the landmarks annotations. The smallest image is 91×288 pixels size, while the biggest one is 1200×1600 pixels size. Every image was manually annotated with 55 landmarks. A sample image and its corresponding landmark points can be observed in Figure 4b.

These datasets mentioned above have been widely used to train and test approaches presented in the literature. Table 1 shows a summary of the number of classes, samples, and samples per class of all different datasets available in the ear recognition context.

## 3. Related Work

In the following paragraphs, we summarize the latest approaches that have been conducted in the unconstrained ear recognition context, especially approaches that implemented DL techniques in their proposals.

One major problem when using deep learning methods and training models to learn representing ears is the lack of enough training data; the following approaches employed techniques like data augmentation and transfer learning and different domain adaptation strategies to cope with this problem. First, [28] applied an aggressive data augmentation process to show the impact in full and selective learning models. Full learning refers to learning from scratch, while selective learning refers to initializing a model with parameters already learned from the ImageNet dataset and fine-tuning specific layers only. The authors used three models in their experiments VGG16, AlexNet, and SqueezeNet. Using 1383 unconstrained ear images from the AWE dataset to train, authors reach a Rank-1 value of 41.26% with no data augmentation. in contrast, with an augmentation factor of 100 in the train set, they obtained 62% Rank-1 applying selective learning in the SqueezeNet over 921 testing images. Second, the author in [29] presented a deep learning pipeline for using transformer neural networks: Vision Transformer (ViT) and Data-efficient image Transformers (DeiTs). Similar to the concept of transfer learning on pretrained state-of-the-art CNN architectures, this study replaced the final layer of ViT and DeiT to enable the transformer network to learn the features from the extracted training ear images of the EarVN1.0 and UERC datasets. Nonetheless, due to the constraint of computational resources, the author considered only the first 20 classes of the EarVN1.0 dataset and the first 10 classes of the UERC dataset. Results report a recognition accuracy of 96.11% on the EarVN1.0 dataset and 100.00% on the UERC dataset.

Finally, one last approach, that uses CNNs and a transfer learning process, is presented in [13]. Slightly different from the two previous approaches, the authors in this work proposed a two-stage fine-tuned process for domain adaptation. In the first stage, authors fine-tune a model using 17,000 ear images from 205 subjects. All images among individuals present the same angle positions and illumination since images were collected for research purposes. in the second stage, the authors perform a final fine-tuning process in the target dataset, in this case, the UERC dataset. Authors present their experiments applied to three models: VGG16, AlexNet, and GoogLeNet. Results show the best performance of VGG16 over the other two models, with an accuracy value of 54.2% employing one-stage fine-tuning while using the two-stage fine-tuning process to increase the accuracy up to 63.62%. They conclude that a two-stage domain adaptation process is definitely necessary and valuable to adjust pretrained models to the ear recognition task.

Experimental results presented in [30] show a comparison among extracted features using handcrafted descriptors and learn features using CNNs. They used seven top handcrafted descriptors and four AlexNet-based CNNs models to evaluate performance extensively. The obtained results demonstrated that CNN features were superior in recognition accuracy, outperforming all handcrafted features. the performance gain in recognition rates was above 22% over the best performing descriptor on the AMI and AMIC datasets, where the number of images per subject was relatively small. However, the performance gain was within 3% for the CVLE dataset, which had fewer subjects and more images per subject, but higher intraclass variability. Even though authors show that CNNs can be trained using small datasets, they point out that it is possible to improve the performance further when more training data is available to learn.

Being aware that both techniques, handcrafted and CNNs, have discriminative powers when using them for ear image description, some other authors look for fusion methods of both techniques. For instance, in [31], the authors first train a CNN-based landmark detector using the In The Wild dataset. The network architecture receives a greyscale image and outputs a 110-dimensional vector that represents the 2D coordinates of 55 landmarks. Then, using the detected landmarks, a geometric normalization is applied among all images in the dataset. On the one hand, feature vectors were obtained using handcrafted techniques such (LBP, BSIF, LPQ, RILPQ, POEM, HOG, and DSIFT). on the other hand, a CNN architecture was trained from scratch. Then after training, a feature extractor was used to obtain a feature vector. Using principal component analysis (PCA), all feature vectors were reduced to the same size. Finally, matching scores from both feature vector types were summed up with “sum fusion“ after applying a min-max normalization process. They validate their study on the AWE dataset in a close-set evaluation, with a 75.6% Rank-1 value, and the UERC dataset Rank-1 49.06% in an open-set evaluation. the best result was given by fusing the CNN and HOG feature vectors in both cases. They conclude that including a normalization step and a fusion of feature vectors increases recognition performance.

Similarly, [32] presented a model called ScoreNet, which includes three basic steps. in the first step, a modular pool of modalities is created. A modality is understood as a single operation in a standard recognition pipeline such as image resizing, preprocessing, image representation (using handcrafted or learned features), dimensionality reduction, and distance measurement. In the next step, modalities are selected randomly for each step in the pipeline generating a random ear recognition pipeline. Then, the pipeline is applied to the training and validation set to generate a similarity score matrix. Once multiple such pipelines are generated, an algorithm called Deep Cascade Score Level Fusion (DCSLF), proposed in that work, selects the best groups of modalities and calculates the necessary fusion weights. In the last step, all selected modality groups (pipelines) and calculated fusion weights in the cascaded network structure are fixed for the test dataset. The authors report a 61.5% regarding Rank-1 performance metric over the UERC dataset. In addition, they show that implementing landmark-based orientation and scale normalization procedures increases overall performance.

Nonetheless, the ScoreNet architecture suffers from long training and diversity in the modality pool. These two last approaches were presented in Unconstrained Ear Recognition Challenge 2019 [25], being the top two techniques that used hybrid approaches (learned and handcrafted) and obtained the best results in the ear recognition task over a set of 1800 images from 180 subjects (10 images per subject). Nonetheless, this approach suffers from long training requirements and a diverse modality pool to find the best fusion options.

Instead of combining different extraction techniques, other researchers build ensembled CNN architectures to enhance recognition accuracy. For example, experiments presented in [14] showed that by assembling different architectures trained on the same dataset, recognition rate improves than using single architectures. In this case, gathering architectures produce a model surgery by replacing pooling layers for spatial pyramid pooling layers to fit arbitrary data size and get multilevel features; authors also implemented center loss to obtain more discriminative features. Experiments were performed over a dataset that authors built for training and testing their approach and the AWE dataset. Similarly, a second study presented in [33] shows improvements by ensembling three ResNeXt101 models. the study shows a comparative analysis between two scenarios, using CNNs for feature extraction only and then training top layers for classification. In contrast, the second scenario included replacing top layers, training them, and fine-tuning the complete architecture. a critical fact that led to a slightly better upgrade in the final results was preserving the aspect ratio of images when training. Thus, according to their experiments, a fine-tune-architectures ensemble trained with custom input size performs best. Experiments and results report rank-1 recognition accuracy above 90% using the EarVN1.0 dataset. Finally, authors in [34] present a similar proposal by building ensembles of ResNets networks of various depths. The best performance is obtained by averaging ensembles of fine-tuned networks achieving recognition accuracy of 99.64%, 98.57%, 81.89%, and 67.25% on the AMI, AMIC, WPUT, and AWE databases, respectively.

Ultimately, some other approaches handled ear recognition by including machine learning techniques and genetic algorithms in their proposals. The authors of [35] proposed a framework based on a hybrid approach of learning distance metric (LDM) and directed acyclic graph (DAG) with support vector machine (SVM). Their proposal aims to learn a Mahalanobis distance metric via SVM to maximize interclass variation and minimize intraclass variation simultaneously. This proposal gets classification accuracy up to 98.79%, 98.70%, and 84.30% for AWE, AMI, and WPUT ear datasets, respectively. Finally, the author in [36] evaluated a complete pipeline for ear recognition using a Faster Region-based CNN (Faster R-CNN) as object detector, a CNN as feature extractor, principal component analysis (PCA) for feature dimension reduction, a genetic algorithm for feature selection, and a fully connected artificial neural network for feature matching. Experimental results show the time needed for the complete pipeline execution, 76 ms for matching (database of 33 ear images features), 15 ms for feature extraction, and ear detection and localization requiring 100 ms. the total time to run the system is 191 ms, which can be used in real-time applications with high accuracy. Experiments were performed on a combined AMI dataset and images taken from the author’s environment.

A summary of the latest approaches is presented in Table 2.

## 4. The VGGFace-Ear Database

The following subsections detail the VGGFace-Ear dataset construction and all the processes performed to collect the final data. We built it from a released face dataset. All the generated data and metadata are available in CSV files (https://github.com/grisellycooper/VGGFace-Ear, accessed on 13 January 2022).

### 4.1. Obtain the Raw Data

The VGGFace2 dataset [12] collects images that were searched and downloaded using Google Image Search and have large variations in pose, age, illumination, ethnicity (including Chinese and Indian people), and profession (e.g., actors, singers, athletes, politicians). It gathers around 3.31 billion images belonging to 9131 subjects, with an average of 363 images per subject. It is approximately gender-balance, containing 41.7% of samples from females. Images are variable-sized and saved as JPG format files. It includes human-verified face bounding boxes and five landmarks on eyes, nose, and mouth corners. the dataset is divided into two sets; the train set gathers 8631 classes, and the test set, 500 classes. Table 3 shows some insights from both sets. This dataset is available at [37].

### 4.2. Detecting Ear on Raw Data

**Figure 5:**
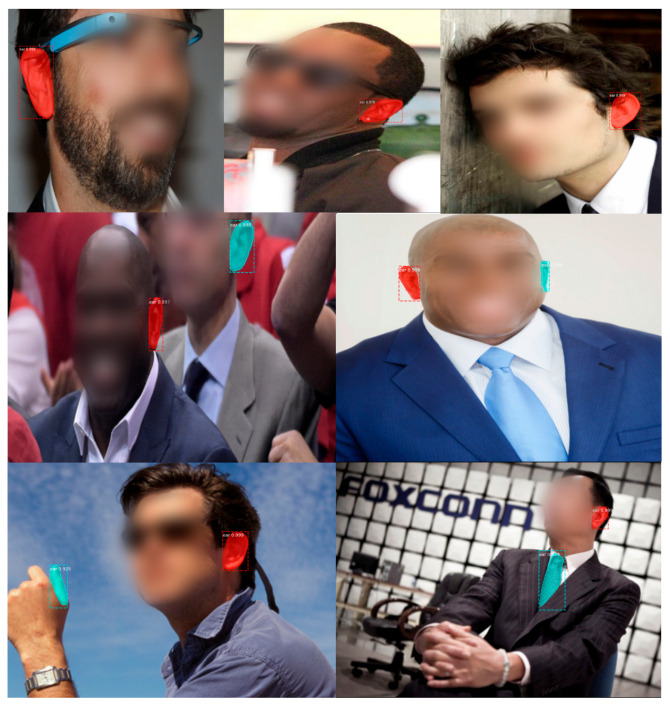
Ear detection using the Mask R-CNN. on the first row, detections of only one object per image, on the second row, it shows detections of two ears from the same person and different people, finally, the last row show also fail detections.

The ear detection task was performed using the Mask-Region-based Convolutional Neural Network (Mask R-CNN) introduced in [38]. Unlike other region-based architectures, this model detects objects at a pixel level, which means that one of the outputs is a binary mask that indicates whether a given pixel is part of an object. The other two outputs are bounding box coordinates and the object class label. In this case, we trained a model using the AWE for Segmentation Dataset [17] and a ResNet101 as a backbone architecture. We performed a fine-tuning process of all layers of a model already pretrained with the ImageNet dataset. Over the 1000 available images, we used 900 images for training and 100 for testing the model. Considering an Intersection over Union (IoU) threshold of 0.5, the Average Precision (AP) value was 97.5%, and considering a threshold of 0.75, the AP was 92.5% over the test set. Even though the model detects ears with high accuracy, it is necessary to apply different filters to obtain “clean” data. Figure 5 shows some ear detection samples.

### 4.3. Automatic Data Filtering

After detection, the number of samples decreased around 28% in the training set. This means that on 28% of the raw images, the model could not detect a single ear object. The number of samples in the test set also dropped approximately 26%. We trained a classifier to distinguish ear from non-ear objects to ensure correct ear detection on every sample. the classifier was built from a fine-tuned ImageNet pretrained CNN with a dataset explicitly constructed for this purpose. Using the classifier’s score or confident value, we analyzed every value range. We saved images with a value between 0.9 and 1.0 which represent 67.8% of all samples. Images between 0.8 and 0.9 represent around 3% of samples. After checking some samples, we observed that most of them were actually ear objects, thus we decided to include this group as well. However, checking samples from 0.7 and 0.8 (around 2%), we encountered some objects that were not actually ears, so we decided not to include this group of samples. In addition, we remove some images that represent outliers regarding size, mask information, and aspect ratio. In these cases, we make decisions after observing samples within each group. First, images with a height or width less than 15 pixels were pulled apart. Maximum dimensions were not set. Second, since information about the mask was available, we used it to get rid of images that did not reach one-third pixels over the total number of pixels of the image. Finally, images with extreme aspect ratios were also discarded, and solely images that fit the range between 0.6 and 3.5 values were saved. Ultimately, these filters diminished the number of samples to approximately 1.3 million images of 8624 classes in the train set and around 72 k samples of 499 classes in the test set.

### 4.4. Manual Filtering

Then we manually performed a second filter over the remaining images. First, images that present more than two objects were checked manually to remove samples where ears detected do not belong to the main person in the picture. In some cases, more than one person appears in the image; thus, some other ears were also detected for a single image. in addition, any other object that was not ear was removed. Finally, one more filter was applied regarding the number of samples. We set a minimum of 250 samples per class to be part of the training set. This reduces samples by around 50% and to 1650 classes. Furthermore, by analyzing samples size and aspect ratios and avoiding classes that have little variability among its samples, only 600 classes were chosen to be part of the final training set. On the other hand, the test set was also filtered by a minimum number of samples, 150. Subjects were chosen to gather the most possible variable set taking into account gender and race/ethnicity information, ending up with 60 subjects in the final test set where 66.7% are male subjects. Table 4 shows statistics of the final sets.

### 4.5. Annotation Files Description

**Figure 6:**
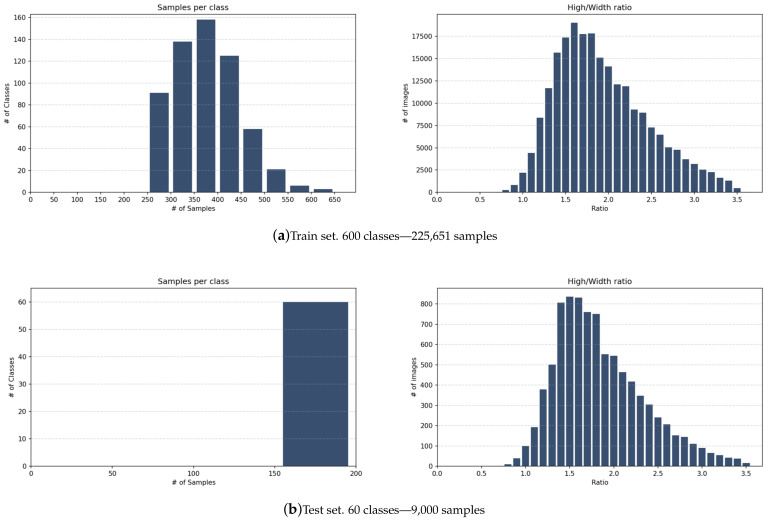
VGGFace-Ear dataset statistics.

All data generated after filtering was exported into CSV files for train and test sets. Annotation files contain data about detection, ear and non-ear classification, and some other calculated values generated to filter images. Table 5 presents a description of the information stored in the annotation files. Two versions of the annotation files are available; the first was generated after the automatic filtering, and the second was after the last filtering. Thus, only the final train and test sets of the VGGFace-Ear dataset were human revised. Figure 6 shows insights of the generated data regarding the number of samples per class and the aspect ratio of all images in the dataset.

## 5. Transfer Learning for Ear Recognition

### 5.1. Pretrained Models

We employed VGG-based models to train ear image representation. First, the VGG16 [39], presented as a submission of the ILSVRC 2014 competition, achieves 92.7% top-5 test accuracy over 14 million images belonging to 1000 classes. This model and others were implemented in most DL frameworks, and also the weights are available for these trained models using the ImageNet Database. This architecture contains 16 layers divided into five convolutional groups and three final dense layers. We changed the input size for our experiments, considering the average height/width ratio of samples in the train set. Also, we added one dense layer of 2622 units before the softmax activation layer, trying to get a lower-dimensional representation for ear images.

Second, the VGGFace [12], trained with the VGGFace2 dataset, has its architecture and weight shared by their authors. The VGGFace2 dataset contains more than 9000 identities with an average of 362.6 images per subject. Images were collected from the Internet using web crawlers. the architecture is similar to the VGG16, with five convolutional groups but three fully connected layers at the top before the final softmax layer, two of 4096 units, one of 2622 units. For our experiments, we used the original input size of 224×224 and did not remove any layer, except for the final activation layer, which was changed to fit the number of classes in the target set. We train and evaluate models using the Tensorflow framework [40].

### 5.2. Transfer Learning

Parameters from both architectures, VGG16 and VGGFace, were fine-tuned to adapt these networks to the ear recognition domain. A fine-tuning process trains the network for more iterations on a target dataset. This way, we avoid computational costs when training deep networks from scratch for new tasks. Also, we leverage the already learn representations on the initial dataset and try to adapt them for a different task. Thus, filters trained on ImageNet and VGGFace2 datasets are adapted to the new VGGFace-Ear dataset. To achieve this, the VGG16 was modified as follows. First, the input size was changed to 112×224 pixels, paying attention to the most common aspect ratio within all images in the dataset. Second, all top dense layers were removed and replaced by four new dense layers, two of 4096 units, one of 2622, and the last layer with 600 units, which is the number of classes in the target dataset. So, these four last layers were trained from scratch while the rest (convolutional layers) were fine-tuned. We removed all dense layers because these units were trained to classify the 1000 objects of the ImageNet. However, for the VGGFace model, the top dense layers remained, and only the last layer was replaced for a new one with 600 units, the number of classes in the VGGFace-Ear dataset. We considered a close relation between face and ear objects in this case, that is why the top layers were also fine-tuned as well as the rest of the layers. Finally, we considered an input size of 224×224, just like the original network. We refer to this last model in the experimental section as the VGGEar model.

## 6. Experimental Results and Discussion

This section presents the experiments using the approach defined in [15]. The results will serve as a baseline for future experiments.

### 6.1. Datasets

We conducted the recognition experiments on three unconstrained ear datasets. First, the proposed dataset, the VGGFace-Ear, gathers 234,651 images belonging to 660 subjects. Samples per subject range between 150 and 645 images. Sample images from this dataset are shown in Figure 3f. Second, the UERC Dataset collects 11,804 samples from 3706 individuals. Samples per subject range from 1 to 153. Images encountered in this set are shown in Figure 3b. Finally, the EarVN1.0 Ear dataset. This collection consists of 28,412 images acquired from 164 subjects. In this case, samples per subject range from 107 to 300 images. Images from this dataset are displayed in Figure 3d.

These three datasets present images collected under uncontrolled conditions. The VGGFace-Ear and the UERC collect images from the Web from public figures and famous people (e.g., athletes, musicians, politicians, actors, actresses, among others) while the EarVN1.0 collects images taken outdoors by camera systems from people in Vietnam during 2018. All images in these sets show high head rotation variations, illumination, occlusion, background variety, race, and gender. in addition, images are different sizes, resolutions, and blur cases are also present. Having many subjects and many samples per subject with high variability guarantees both high inter-class and intra-class diversity. These sorts of characteristics make ear recognition a very challenging task.

### 6.2. Experimental Protocols

We split the VGGFace-Ear into two disjoint sets for our analysis and recognition experiments. the first set used for training contains 600 classes and the second set, used for testing, comprises 60 classes. Then, the training set was divided again into train and validation sets. Instead of setting a specific percentage of images per class to train, we took a specific number of samples from every class to validate. the remaining samples were augmented to reach the same number for every class and used to train models. Since classes contain 250 samples or above, we randomly chose 80 samples from every class for the validation set. Remainder samples were applied data augmentation techniques to reach 900 samples per class and used for training. Both VGG16 and VGGEar models were fine-tuned using the train and validation sets. All layers were set trainable and trained using an SGD optimizer for 50 epochs. The learning rate was initially set to 0.001 and then decreased gradually according to how the learning process was evolving.

For UERC dataset, we follow an experimental protocol as in [25]. The authors provided train and test splits to train and test the models. In this case, we applied a two-stage fine-tuning process. The already fine-tuned VGGEar model (using the VGGFace-Ear dataset) was adjusted one more time to a new target dataset, the UERC. Using the training set of 166 subjects, most of which contain ten samples per class, we used four samples for validation, and the rest were augmented to 100 samples per class and used for training. Regarding training, four convolutional groups from the model were frozen, the top layer was changed to fit the new number of classes in the UERC train set, and the learning rate was set from 0.0001 to 0.00001. We adjusted the model for around 80 more epochs.

Concerning the EarVN1.0, we split the dataset into two disjoint sets, holding 60% and 40% of ear images for the train and test set, respectively. We follow this setup to compare experiments with [33]. In this case, we also applied a two-stage fine-tuning process to adapt the already fine-tuned VGGEar model to the EarVN1.0 dataset, just as it was done for the UERC dataset. However, since this collection contains more samples per class, we took 50 samples for validation, and the rest was augmented to 250 samples to train. The model was also adjusted for 80 more epochs.

### 6.3. Data Augmentation and Preprocessing

**Figure 7:**
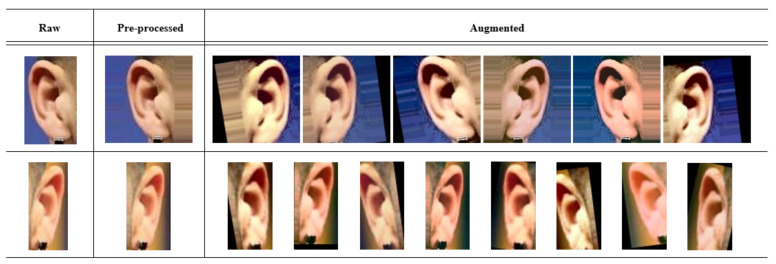
Preprocess and data augmentation examples. First row for the case of square input size (224×224), second row for the 1:2 aspect ratio (112×224).

Some common data augmentation techniques were applied, such as scale in both axes (−20%, 20%), translation in the x-axis (−20%, 20%) and y-axis (−10%, 10%), rotation (−15, 15), brightness and contrast increasing, and decreasing, histogram equalization, and vertical flipping. Besides data augmentation, we also preprocessed samples using some common image preprocessing methods to fit different architecture’s input sizes: resizing, cropping, and filling. The resizing may result in geometric distortion, and cropping may discard important information even more, when images are so small. Finally, filling with the nearest or adding a background extended from the border pixels of the image is our choice. With this technique, we prevent distortion and losing information. We preprocessed images to be squared for the VGGEar and VGG16 models, and images were fit the aspect ratio of 1:2. Figure 7 show instances for both, preprocessing and data augmentation.

### 6.4. Recognition and Feature Extraction

To perform recognition on known identities (close-set recognition), we use predicted classes from the models and asses with accuracy values on train and validations sets. While for recognition of unknown classes or identities (open-set recognition), we use the models as feature extractors and then apply cosine distances to find out the closest class and set it as predicted. in this case, we consider fully connected layers as feature vectors. Unknown classes were predicted by selecting samples from the gallery which feature vector is the closest to the probe sample in an all-vs-all comparison.

### 6.5. Performance Metrics

To present, analyze and assess recognition performance using the proposed dataset, we report our experimental results using the following metrics:

- *Rank-1* refers to the fraction of probe images for which an image of the gallery is correctly retrieved in the top match.
- *Rank-5* refers to the fraction of probe images for which an image of the gallery is correctly retrieved within the top-five matches.
- *Cumulative Matching Curve (CMC)* is a Rank-based curve showing the probability that a model will return the correct identity within the top *k* ranks, where *k* varies from 1 to *N*, being *N* the number of classes in the whole set.
- *Area Under CMC Curve (AUCMC)*, based on CMC, the area under the curve is calculated.

Besides metrics, we utilize a dimensional reduction and visualization technique named t-SNE [41] to depict features generated by the models. It will reveal the ability of the network to discriminate samples from different subjects.

### 6.6. Experimental Results

**Figure 8:**
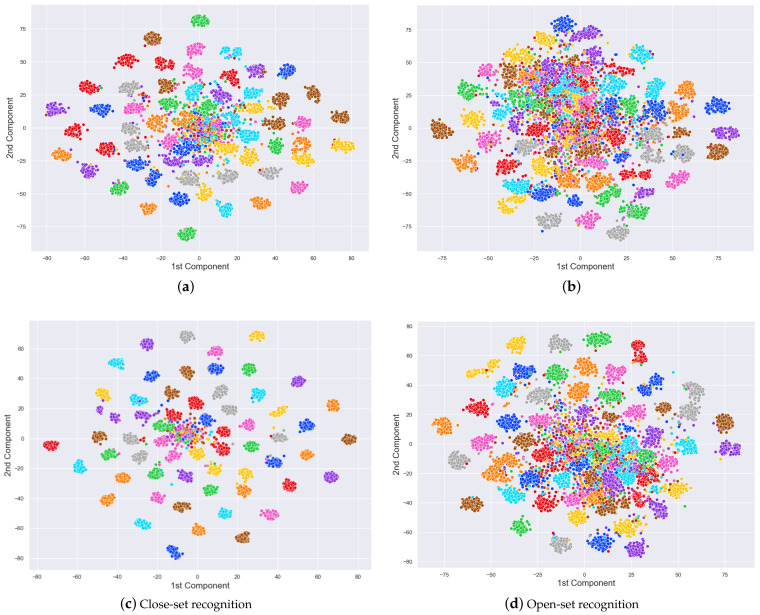
Using the t-SNE algorithm [41], 2D embedded feature vectors were generated for the VGGFace-Ear validation and test sets using the penultimate layer of both models. The first row depicts feature vectors using the VGG16 while the second row, feature vectors using the VGGEar. Every color cluster represents a class in the sets. (**a**) validation set with 80 samples per class and (**b**) test set with 150 samples. The number of classes was reduced to 60 for better visualization. Images elaborated by the author.

We have trained models using the proposed dataset and present assessment within a series of tables and figures. First of all, Table 6 shows a general performance of the two fine-tuned models over the train, validation, and test sets from the VGGFace-Ear dataset. Even though the VGG16 model seems to overfit the train set, both models show generalization capability to unseen data. Considering challenging samples present in the dataset as well as unknown identities which were not part of the train set. This generalization can also be visualized in Figure 8b, where 2622-dimensional feature vectors from the test set using both models were embedded into a 2-dimensional space. Despite challenging samples present in the dataset, models can discriminate identities from 60 different people. Figure 8a depicts feature vectors for the validation set (identities known by the model), while Figure 8b show feature vectors for the test set (identities unknown by the model). For better visualization, only 60 identities are considered in both figures.

**Figure 9:**
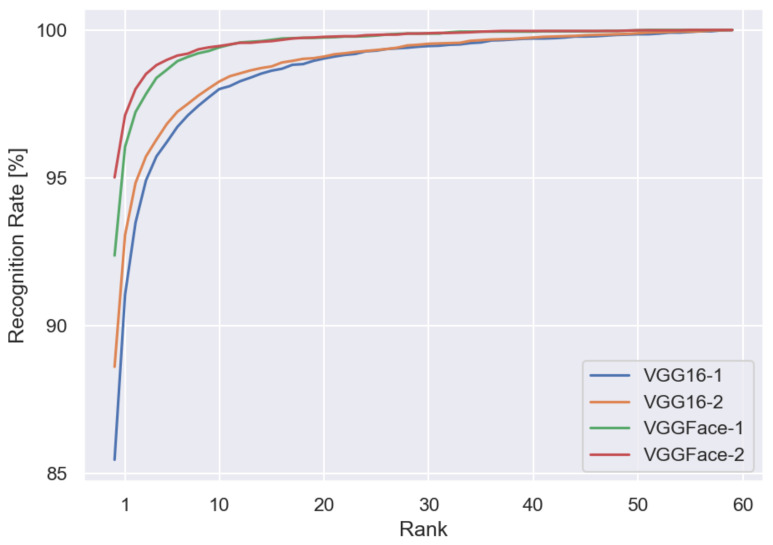
CMC curves for the test set of the VGGFace-Ear dataset.

We also considered different feature vectors from different models’ layers. Table 7 shows the recognition rate from both models with different fully connected layers as feature vectors. We observed that in the case of the validation set, the recognition rate reaches a better performance by using the 2622-dimensional layer, the one before the classification layer; however, in the case of the test set, better performance is achieved using a previous layer, the 4096-dimensional one. Since we are interested in using the model as a feature descriptor to predict unknown identities using similarity, we use the 4096-dimensional layer for the rest of the experiments. Figure 9 shows CMC from the two fine-tuned models and the two different dimensional feature vectors.

Finally, we compare our fine-tuned models with state-of-the-art approaches. For these experiments, we use train sets from both datasets, UERC and EarVN1.0, to perform a second fine-tune of the VGGEar model, as is explained in the experimental protocols section.

Table 8 shows comparative results using the UERC Dataset. As it can be noted, our approach outperforms previous works. We want to point out that previous works tested on the UERC dataset include promising approaches using different CNN architectures, handcrafted techniques, and even fusion techniques or ensembled models that, according to [25], produce better results than using only one technique. However, most previous approaches used the limited training sets available at that time, which collected constrained samples and few samples per person. Even applying aggressive data augmentation techniques and using pretrained models, it was difficult to perform well since CNN demands large amounts of training data and high variability among classes and samples from the same class. Hopefully, the proposed dataset will help solve the unconstrained ear recognition problem.

Table 9 shows comparison using the EarVN1.0 dataset. in this case, we are comparing different strategies that have been proposed in the literature to approach this dataset specifically. Even though we do not achieve a better performance than literature approaches, using simpler models, we achieve competitive recognition rate results compared to the best ones reported in [33].

## 7. Conclusions and Future Work

This work introduces a new large-scale image collection for ear recognition called the VGGFace-Ear dataset. We leverage an existing face dataset to detect ear images and extend the dataset to the unconstrained ear recognition problem. We describe all the processes we go through to build the dataset and save files annotations to make it available for other researchers. Sample images present variability in illumination, head rotation, size, image resolution, and occlusion, ensuring high inter-class and intra-class variability. Furthermore, pretrained models on image and face recognition were used with the proposed dataset to adapt these models to the ear recognition domain. We test the models using two different unconstrained ear datasets. Even though the models used are simple, we obtained a CNN-based feature descriptor with good generalization capability to cluster images using similarity.

Nonetheless, improvements remain, and some other aspects should be addressed. First, any sort of landmark detection, alignment, or normalization may help a CNN focus more on the ear structure features than pose variations. Using pose and age annotation from VGGFace could help get a more homogeneous variable dataset besides the image aspect ratio and include more female samples. Finally, using the proposed dataset to train more complex and deeper architectures like ResidualNets may improve the recognition rate. 

## Figures and Tables

**Table 1:** Overview of available ear datasets in the literature.

Datasets	Classes	Samples per Class	Total Images
UBEAR [16]	126	34–36	4430
AWE [17]	100	10	1000
IITD [19]	125	3–5	493
AMI [20]	100	7	700
WPUT [21]	501	4–8	2070
UERC [25]	3706	1–153	11,804
EarVN1.0 [26]	164	107–300	28,412
In the Wild [27]	231	1–14	2058
VGGFace-Ear (Proposed)	660	150–645	234,651

**Table 2:** Summary of the latest ear recognition approaches.

Approach	Technique	Metric	Dataset	Value [%]
[28]	CNN	Rank-1	AWE + CVLE	61.9
[29]	Transformer	Accuracy	UERC EarVN1.0	100.0 96.1
[13]	CNN	Accuracy	UERC	63.6
[30]	CNN	Rank-1	AMI AMIC CLVE	94.5 89.8 87.1
[31]	CNN + Handcrafted	Rank-1	AWE UERC	75.6 49.1
[32]	CNN + Handcrafted	Rank-1	UERC	61.5
[14]	Assembled CNNs	Accuracy	USTB-HelloEar	90.1
[33]	Assembled CNNs	Rank-1	EarVN1.0	95.8
[34]	Assembled CNNs	Accuracy	AMI AMIC WPUT AWE	99.64 98.57 81.89 67.25
[35]	LDM-DAGSVM	Accuracy	AWE AMI WPUT	98.8 98.7 84.4
[36]	CNN + PCA + Genetic Algorithm + ANN	Accuracy	AMI + Own	97.7

**Table 3:** Quantitative information about VGGFace2 train and test sets.

	Train Set	Test Set
Number of classes	8631	500
Total number of samples	3,141,890	169,396
Mean of samples per class	364	339
Minimum number of samples in a class	87	90
Maximum number of samples in a class	843	761
Mean ratio rate of all samples	1.1	1.1
Mean height of all samples	257.8	257.3
Mean width of all samples	241.0	238.8
Smallest size among all samples	32×31	26×30
Biggest size among all samples	4316×3456	3590×3744

**Table 4:** Quantitative information about the generated VGGFace-Ear dataset.

	Train Set	Test Set
Number of classes	600	60
Total number of samples	225,651	9000
Mean of samples per class	376	150
Minimum number of samples in a class	252	150
Maximum number of samples in a class	645	150
Mean ratio rate of all samples	1.9	1.8
Mean height of all samples	68.6	64.6
Mean width of all samples	36.8	36.1
Smallest size among all samples	15×15	17×19
Biggest size among all samples	707×861	250×514

**Table 5:** Annotation data description.

Field	Type	Description
folder	string	Class name given by the VGGFace dataset
file_img	string	Image or sample name given by the VGGFace datase
num_object	int	Order number of ear detected in the image using the Mask-RCNN
score	float	Detection score given by the Mask-RCNN
y	int	Y Upper-left value of the bounding box given by the Mask-RCNN
x	int	X Upper-left value of the bounding box given by the Mask-RCNN
y2	int	Y Lower-right value of the bounding box given by the Mask-RCNN
x	int	X Lower-right value of the bounding box given by the Mask-RCNN
height	int	Calculated height given bounding box coordinates
width	int	Calculated width given bounding box coordinates
ratio	float	Calculated aspect ratio given height and width
mask per	float	Calculated percentage of pixels of the mask over the total number of pixels in the image
ear_nonear	float	Classification score from the ears and non-ears classifier

**Table 6:** Overall performance of the two fine-tuned models.

Model	Train	Validation	Test
	Accuracy [%]		Rank-1 [%]
VGG16	99.99	90.03	92.89
VGGEar	99.94	95.59	95.00

**Table 7:** Performance evaluation of the two fine-tuned models over the VGGFace-Ear test set.

Model	Feature Vector Size	Rank-1 [%]	Rank-5 [%]	AUCMC [%]
VGG16	4096 2622	92.89 90.24	98.48 97.91	97.90 97.79
VGGEar	4096 2622	95.00 92.37	98.81 98.38	97.99 97.90

**Table 8:** Comparative evaluation with methods in the state-of-the-art over the UERC dataset test set. Bold values indicate the best performance of all different approaches for each metric.

Approach	Rank-1 [%]	Rank-5 [%]	AUCMC [%]
Fusion CNN-HOG [31]	49.06	69.94	95.10
SqueezeNet [28]	62.00	80.35	95.51
ScNet-5 [32]	61.50	80.89	97.10
VGG-16 [13]	63.60	-	-
Fusion VGG16-VGGFace [15]	75.30	89.10	98.10
VGGEar (Ours)	**82.22**	**93.83**	**98.67**

**Table 9:** Comparative evaluation with methods in the state-of-the-art over the EarVN1.0 dataset test set.

Strategy	Architecture	Rank-1 [%]	Rank-5 [%]	AUCMC [%]
Fine-tuned + square-sized inputs [33]	InceptionV3	90.40	97.35	99.06
Fine-tuned + square-sized inputs (Ours)	VGG-based	92.58	97.88	97.61
Fine-tuned + custom-sized inputs [33]	ResNetXt101	93.45	98.42	99.18
